# Bringing together but staying apart: decisive differences in animal and fungal mitochondrial inner membrane fusion

**DOI:** 10.1111/brv.13168

**Published:** 2024-11-18

**Authors:** Hassan Hashimi, Ondřej Gahura, Tomáš Pánek

**Affiliations:** ^1^ Institute of Parasitology, Biology Centre Czech Academy of Sciences Branišovská 31 České Budějovice 370 05 Czechia; ^2^ Department of Molecular Biology and Genetics, Faculty of Science University of South Bohemia Branišovská 31 České Budějovice 370 05 Czechia; ^3^ Department of Zoology, Faculty of Science Charles University Viničná 7 Prague 2 128 00 Czechia

**Keywords:** apoptosis, cristae, dynamin‐related protein, membrane remodelling, mitochondrial dynamics, Mgm1, Opa1, phylogeny

## Abstract

Mitochondria are dynamic and plastic, undergoing continuous fission and fusion and rearrangement of their bioenergetic sub‐compartments called cristae. These fascinating processes are best understood in animal and fungal models, which are taxonomically grouped together in the expansive Opisthokonta supergroup. In opisthokonts, crista remodelling and inner membrane fusion are linked by dynamin‐related proteins (DRPs). Animal Opa1 (optical atrophy 1) and fungal Mgm1 (mitochondrial genome maintenance 1) are tacitly considered orthologs because their similar mitochondria‐shaping roles are mediated by seemingly shared biochemical properties, and due to their presence in the two major opisthokontan subdivisions, Holozoa and Holomycota, respectively. However, molecular phylogenetics challenges this notion, suggesting that Opa1 and Mgm1 likely had separate, albeit convergent, evolutionary paths. Herein, we illuminate disparities in proteolytic processing, structure, and interaction network that may have bestowed on Opa1 and Mgm1 distinct mechanisms of membrane remodelling. A key disparity is that, unlike Mgm1, Opa1 directly recruits the mitochondrial phospholipid cardiolipin to remodel membranes. The differences outlined herein between the two DRPs could have broader impacts on mitochondrial morphogenesis. Outer and inner membrane fusion are autonomous in animals, which may have freed Opa1 to repurpose its intrinsic activity to remodel cristae, thereby regulating the formation of respiratory chain supercomplexes. More significantly, Opa1‐mediated crista remodelling has emerged as an integral part of cytochrome c‐regulated apoptosis in vertebrates, and perhaps in the cenancestor of animals. By contrast, outer and inner membrane fusion are coupled in budding yeast. Consequently, Mgm1 membrane‐fusion activity is inextricable from its role in the biogenesis of fungal lamellar cristae. These disparate mitochondrial DRPs ultimately may have contributed to the different modes of multicellularity that have evolved within Opisthokonta.

## I. INTRODUCTION

Mitochondrial networks are constructed and dismantled by fission and fusion of these organelles (Pernas & Scorrano, 2016) collectively termed “mitochondrial dynamics”. A balance of these two processes is necessary for the upkeep and inheritance of mitochondria. The fusion of two juxtaposed membranes from separate mitochondria is mediated by mechanochemical GTPases that belong to dynamin‐superfamily proteins (DSPs) (Ramachandran & Schmid, 2018). Some DSPs can be subcategorized as dynamin‐related proteins (DRPs) due to their structural similarity to this enzyme (Ford & Chappie, 2019). Mitochondria are enveloped by both an outer (OM) and inner (IM) membrane, with the fusion of each driven by dedicated DSPs. In vertebrate mitochondrial fusion, two OM DSPs called mitofusin 1 (Mfn1) and 2 (Mfn2) work in concert with the IM DRP termed optical atrophy 1 (Opa1) (Pernas & Scorrano, 2016). The OM DSP fuzzy onion (Fzo1) and IM DRP mitochondrial genome maintenance 1 (Mgm1) adopt these roles in ascomycete fungi.

Most knowledge about mitochondrial dynamics comes from animal and fungal model organisms, which belong to the same eukaryotic supergroup Opisthokonta (Burki *et al*., 2020). We discuss herein several lines of evidence for greater differences between the molecular machineries responsible for mitochondrial membrane fusion in animals and fungi than are generally assumed. Animals and fungi have experienced long independent evolution as members of Holozoa and Holomycota, respectively, the two main subdivisions of Opisthokonta (Burki *et al*., 2020; Ocaña‐Pallarès *et al*., 2022). We speculate that distinct properties emerged in animals (Ros‐Rocher *et al*., 2021) and fungi (Nagy, Kovács & Krizsán, 2018) ultimately predisposing each towards different manifestations of multicellularity (Ocaña‐Pallarès *et al*., 2022).

**Fig. 1:**
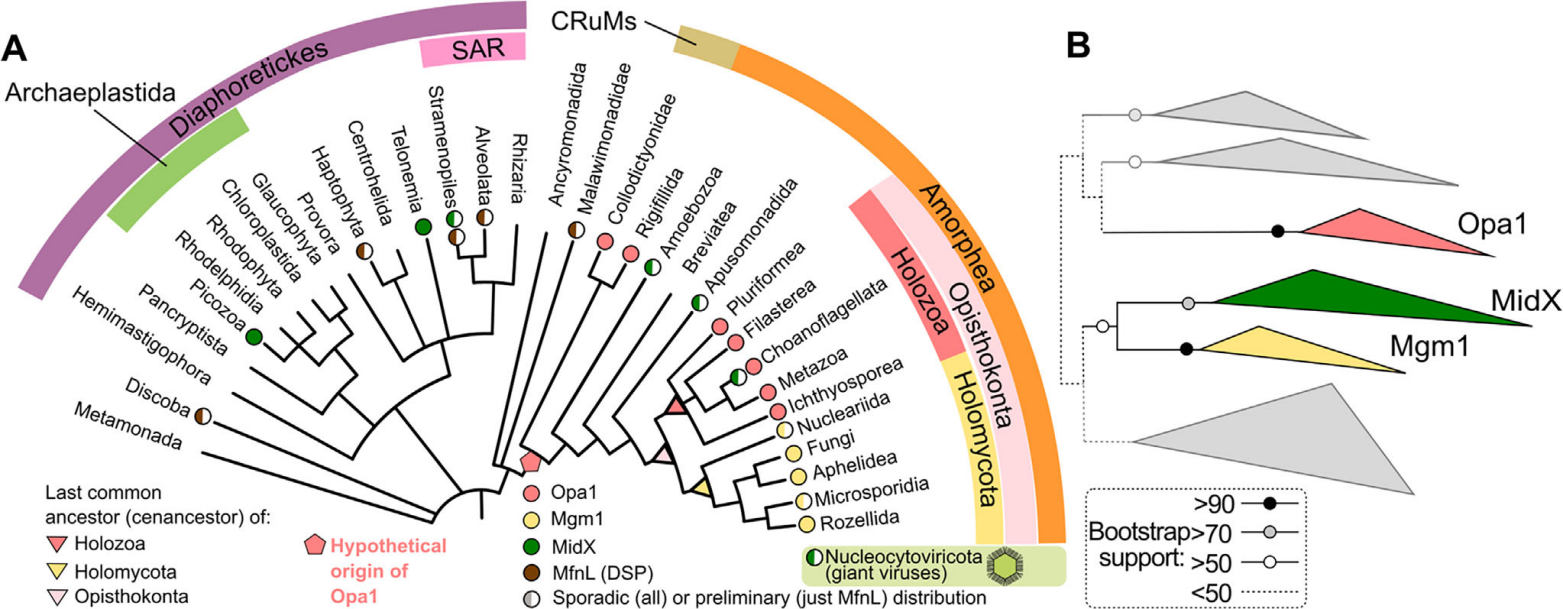
Distribution and phylogenetic relationships among the mitochondrial dynamin‐related proteins (DRPs) Opa1, Mgm1 and MidX. Modified from Sheikh *et al*. (2023). (A) Distribution of the DRPs Opa1, Mgm1 and MidX plus the preliminary and likely incomplete distribution of the inner membrane (IM) dynamin‐superfamily protein (DSP) MfnL (see Section VI.2.a), as indicated by coloured circles (see key on bottom right). The cenancestors of different taxa are indicated by coloured triangles (key on bottom left). A possible origin of Opa1 is indicated by the pentagon. (B) The phylogenetic relationships among Opa1, Mgm1, MidX, and other DRPs (different classes collapsed into grey triangles). Mgm1, mitochondrial genome maintenance 1; MfnL, mitofusin like; MidX, mitochondrial DRP of unknown function; Opa1, optical atrophy 1.

Our thesis hinges on the divergence of the IM fusion DRPs, Opa1 and Mgm1. These plausibly underlie critical differences in mitochondrial dynamics, which ultimately may have contributed to the distinct evolutionary paths taken by animals and fungi. Given the uncanny commonalities between Opa1 and Mgm1 and their placement in two sister groups within Opisthokonta (Fig. 1A), the literature has long ascribed them as direct orthologs, that is originating from a single gene present in their cenancestor, the last common ancestor of both groups (e.g. Griparic *et al*., 2004). The structures of Opa1 and Mgm1 resemble each other to a greater extent than other DRPs (Sheikh *et al*., 2023; Faelber *et al*., 2019; Nyenhuis *et al*., 2023; von der Malsburg *et al*., 2023; Yan *et al*., 2020), reinforcing this view. Indeed, they are more similar to each other than to other IM‐shaping DSPs (Morel *et al*., 2023; Vanwalleghem *et al*., 2015), such as the mitochondrial matrix‐targeted MidX (mitochondrial DRP of unknown function), a *bona fide* DRP encoded in the genomes of giant viruses and six diverse eukaryotic taxa (Sheikh *et al*., 2023). However, recent phylogenetic analyses reliably placed Mgm1 and MidX in the same clade phylogenetically distant from Opa1 (Fig. 1B) [see Sheikh *et al*. (2023) for further details and an in‐depth discussion of the molecular phylogeny employed]. Thus, while Opa1 and Mgm1 are both IM‐remodelling DRPs, they possibly have separate evolutionary origins and evolved convergently.

## II. THE SHORTLISTED CONVERGENT PROPERTIES OF OPA1 AND MGM1

**Fig. 2:**
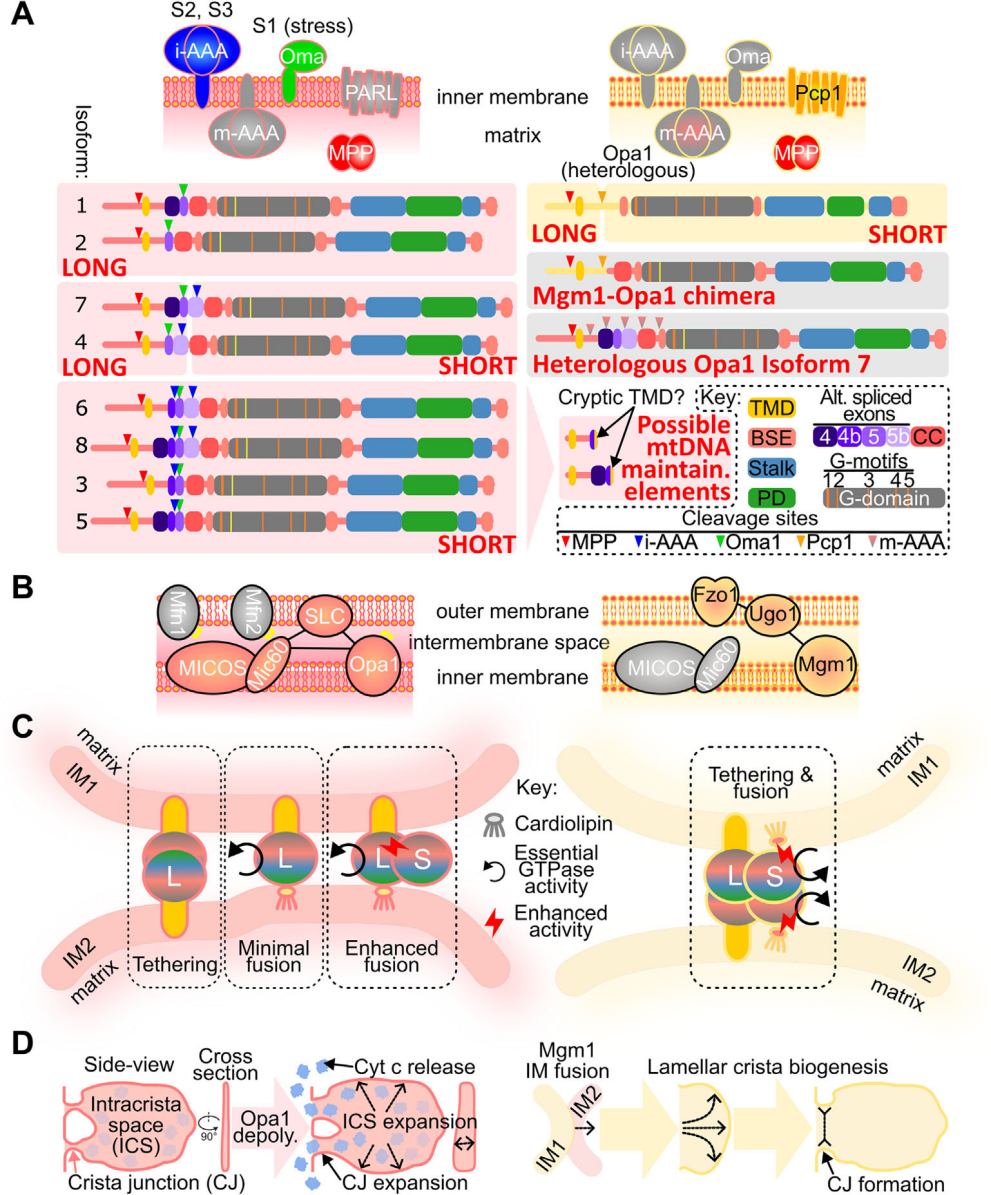
Different properties of Opa1 and Mgm1. (A) Isoform generation mechanisms of Opa1 and Mgm1. The upper two images depict the four proteases that have been implicated in processing each dynamin‐related protein (DRP) in animals and yeast; grey colour indicates no evidence for direct processing. Below left, products of different *opa1* messenger RNA (mRNA) isoforms grouped by their capacity to generate exclusively long (isoforms 1 and 2), long and short (4, 7) and exclusively short forms (3, 5, 6, 8); the N‐terminal part of Opa1 after S3 cleavage proposed to be important for mitochondrial DNA (mtDNA) maintenance. Below right, mRNA isoform with yellow background is endogenous Mgm1 and those with the grey background are Opa1 DRP variants heterogeneously expressed in yeast with estimated m‐AAA cleavage sites in Opa1 shown by pink arrowheads. On bottom right is key to Opa1 exons, shared features of each protein's DRP module and cleavage sites. (B) Interaction network of Opa1 (left) and Mgm1 (right) and other not directly interacting functionally related proteins (grey). Redox motifs of Mfn1, Mfn2 and Opa1 are indicated in yellow. (C) Mechanisms of inner membrane (IM) fusion mediated by Opa1 (left) and Mgm1 (right). Proven essential GTPase activities and enhancements of activities are shown according to the central key. The DRP module is depicted by circle with colours of DRP motifs as in A. L, long form; S, short form. (D) Mechanisms of crista remodelling mediated by Opa1 (left) and Mgm1 (right). Opa1 depolymerization (depoly.) may induce an expansion of crista junctions (CJs) and the intracrista space (ICS) to allow the release of sequestered cytochrome c (Cyt c). By contrast, Mgm1‐mediated IM fusion allows it to facilitate the biogenesis of lamellar cristae. BSE, bundle signalling element; Fzo1, fuzzy onion 1; i‐AAA, intermembrane space‐ATPases associated with diverse cellular activities; m‐AAA, matrix‐ATPases associated with diverse cellular activities; Mfn, Mitofusin; Mgm1, mitochondrial genome maintenance 1; MICOS, mitochondrial contact site and cristae organizing system; MPP, mitochondrial processing peptidase; Opa1, optical atrophy 1; PARL, PINK1/PGAM5‐associated rhomboid‐like protease; PD, paddle domain; SLC, solute carrier; TMD, transmembrane domain.

Opa1 and Mgm1 are sorted to the IM *via* an N‐terminal mitochondrial pre‐sequence and a proximate transmembrane domain (TMD) (Fig. 2A). Consequently, each is membrane‐embedded with their analogous DRP modules extending into the intermembrane space (IMS). After pre‐sequence removal, a fraction of each DRP is proteolytically cleaved before the DRP module to yield a mixed population of integral membrane long (L‐) and soluble short (S‐) forms (Herlan *et al*., 2003; McQuibban, Saurya & Freeman, 2003; Cipolat *et al*., 2006; Ishihara *et al*., 2006).

Ostensibly, Opa1 and Mgm1 play the same roles in mitochondrial dynamics, that is they join separate mitochondria by facilitating IM fusion (Sesaki *et al*., 2003; Meeusen *et al*., 2006; Cipolat *et al*., 2004; Song *et al*., 2009) and remodel IM‐connected cristae (Meeusen *et al*., 2006; Harner *et al*., 2016; Olichon *et al*., 2003; Frezza *et al*., 2006; Cipolat *et al*., 2006). To fulfil both roles, they oligomerize (Faelber *et al*., 2019; von der Malsburg *et al*., 2023; Nyenhuis *et al*., 2023; Yan *et al*., 2020) and cooperate with the IM phospholipid, cardiolipin (DeVay *et al*., 2009; Ban *et al*., 2017). Mitochondrial fusion requires the coordination of IM and OM fusion; thus, the respective animal and fungal IM DRP/OM DSP dyads Opa1/Mfn1 (Cipolat *et al*., 2004; Song *et al*., 2009) and Mgm1/Fzo1 (Meeusen *et al*., 2006) are interdependent. Finally, both DRPs are required for mitochondrial genome (mtDNA) maintenance (Sesaki *et al*., 2003; Elachouri *et al*., 2011; He *et al*., 2022; Jones & Fangman, 1992).

## III. A LONG TREATISE ON THE MECHANISTIC DIFFERENCES BETWEEN OPA1 AND MGM1

Before we disentangle the aforementioned commonalities that have tied the animal and fungal DRPs together in the literature, we first provide a few caveats. Firstly, while Mgm1 and Opa1 have been studied in other animal (Del Dotto & Carelli, 2021; Kanazawa *et al*., 2008; Yarosh *et al*., 2008) and fungal (Leroy *et al*., 2010; Liang *et al*., 2018) models, we restrict most of our discussion to the exhaustive functional data obtained from mammals (human and mouse) and budding yeast (*Saccharomyces cerevisiae*). Secondly, because Opa1 and Mgm1 deletions result in eventual mtDNA loss, we compare phenotypes from induced ablation of the DRPs whenever possible and appropriate to concentrate on their respective direct effects on mitochondrial morphology. Thirdly, we utilize studies that can be directly compared. In accordance, given the elaborate proteolytic cascade that regulates Opa1 (Deshwal, Fiedler & Langer, 2020), we regrettably exclude elegant studies utilizing protease ablation (e.g. Merkwirth *et al*., 2008; Richter *et al*., 2019) as equivalent approaches are not possible in *S. cerevisiae*.

### (1) Isoform biogenesis

While existing as L‐ and S‐forms in both yeast and mammals, the mechanism of Opa1 and Mgm1 isoform generation differs radically. For Opa1, alternative splicing of its transcript produces eight messenger RNA (mRNA) variants (Song *et al*., 2007; Delettre *et al*., 2001) (Fig. 2A), ultimately affecting their proteolytic processing. By contrast, the *mgm1* transcript lacks introns like most budding yeast mRNAs (Ast, 2004), and therefore, the presence or absence of alternative splicing does not provide evidence that Opa1 and Mgm1 are not orthologous.

Opa1 processing involves three cleavage sites between the TMD and DRP module (Fig. 2A) and two integral IM proteases named Oma1 and the i‐AAA protease Yme1L (Anand *et al*., 2014). The Oma1 cleavage site S1 is encoded by exon 5, which is present in all *opa1* mRNAs (Ehses *et al*., 2009; Head *et al*., 2009). Stress (e.g. mitochondrial depolarization) causes Oma1 to cleave every L‐Opa1 into S‐Opa1, leading to mitochondrial network fragmentation (Fry *et al*., 2024; Head *et al*., 2009; Ehses *et al*., 2009). Yme1L, which faces the IMS side from the IM (Deshwal *et al*., 2020), recognizes two Opa1 cleavage sites. Splice variants with exon 5b encode the S2 cleavage site, intermittently cut to produce a mixture of L‐ and S‐Opa1 (Song *et al*., 2007; Griparic, Kanazawa & van der Bliek, 2007). Polypeptides with the exon 4b‐encoded S3 cleavage site are constitutively processed into S‐Opa1 by Yme1L (Wang *et al*., 2020) (Fig. 2A).

Mgm1 proteolytic cleavage is much simpler (Fig. 2A). Its single cleavage site is processed by the rhomboid protease Pcp1, the yeast ortholog of mammalian PARL1 (Herlan *et al*., 2003; McQuibban *et al*., 2003). Interestingly, neither Mgm1 nor Opa1, when heterologously expressed in *S. cerevisiae*, is processed by Yme1L or Oma1 (Duvezin‐Caubet *et al*., 2007). Instead, heterologously expressed Opa1 is cleaved in budding yeast by m‐AAA protease, perhaps mistaken for its cognate substrate, misfolded/damaged proteins (Deshwal *et al*., 2020). Accordingly, Opa1 fails to complement *mgm1* gene deletion (Duvezin‐Caubet *et al*., 2007). Partial complementation is achieved only by fusion of the Mgm1's N‐terminus up to its single cleavage site to Opa1's DRP module to create a Mgm1–Opa1 chimera (Nolli *et al*., 2015) (Fig. 2A). Tellingly, however, mitochondria remain mostly fragmented after complementation with this chimera. These results demonstrate that Opa1 co‐evolved with its processing proteases Yme1 and Oma1 in Metazoa and perhaps all Holozoa. In summary, orthology of Opa1 and Mgm1 is challenged because they are processed by different enzymes from a mitochondrial protease repertoire common to Holozoa and Holomycota.

### (2) Monomeric structure

**Fig. 3:**
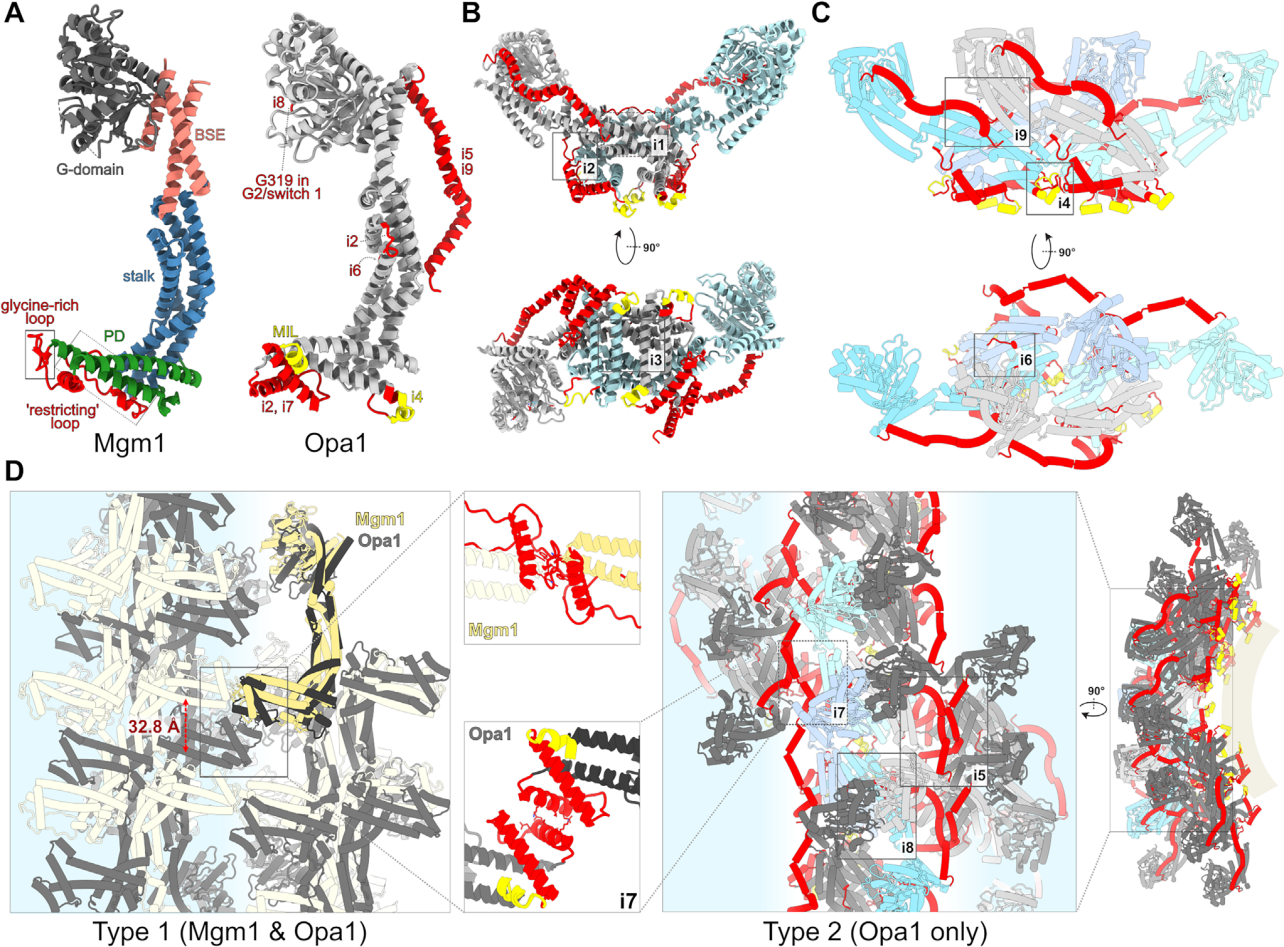
Structural differences between mitochondrial genome maintenance 1 (Mgm1) and optical atrophy 1 (Opa1) affect their modes of oligomerization. (A) Structures of monomeric units of *C. thermophilum* Mgm1 (PDB 6RZV) (Faelber *et al*., 2019) and human Opa1 (PDB 8EFF) (Nyenhuis *et al*., 2023). Regions of Mgm1 are labelled and coloured according to the scheme in Fig. 2A. Elements specific for each dynamin‐related protein (DRP) are shown in red and those of Opa1 that are inserted in the membrane are shown in yellow. The Opa1‐specific elements involved in interfaces in type 2 tetramers and oligomers are labelled i2–i9 (interface nomenclature according to Nyenhuis *et al*., 2023). MIL, membrane insertion loop. (B, C) Positions of interfaces in type 2 dimers (B) and tetramers (C) of human Opa1 (PDB 8EFF) (Nyenhuis *et al*., 2023). Each protomer is coloured individually (in blue/grey shades) and some specific elements are coloured as in A. (D) Oligomeric arrangements of Mgm1 and Opa1. Alternating white and blue background indicates individual rungs. Type 1 arrangement (left) was observed in both Mgm1 (Faelber *et al*., 2019) and Opa1 (von der Malsburg *et al*., 2023). While the overall network is similar, the inter‐rung contacts are mediated solely by paddle domain (PD) elements specific for each DRP (close‐up views), resulting in a ~33 Å shift in the rung alignment between Mgm1 and Opa1. Type 2 has been observed only in Opa1 (Nyenhuis *et al*., 2023) and its formation depends on multiple interfaces, shown also in B and C, mostly formed by Opa1‐specific elements. The only interface shared between both types is i7. Boxes highlight motifs (A) or interfaces (B–D); those in dashed lines highlight obscured motifs or interfaces on the opposite side of the structure.

The structures of S‐Opa1 and S‐Mgm1 conform to the modular organization that typifies all DRPs (Ramachandran & Schmid, 2018; Ford & Chappie, 2019). The GTPase domain (G‐domain) is attached to the DRP's stalk *via* the bundle signalling element (BSE), comprised of three discontiguous α‐helices. Usually, an insertion is found between the stalk's last two α‐helices that represent the paddle (or variable) domain (PD), which mediates most interactions with the membrane (Figs 2A and 3A). The interfaces that allow self‐assembly into homo‐oligomers, an important process for their membrane remodelling activity, are distributed throughout the DRP.

Compared to other DRPs, S‐Opa1 and S‐Mgm1 share a high degree of structural correspondence (Faelber *et al*., 2019; Nyenhuis *et al*., 2023; von der Malsburg *et al*., 2023; Yan *et al*., 2020; Sheikh *et al*., 2023). However, closer scrutiny reveals apparent differences between them, which determine fundamental biochemical – and ultimately mechanistic – disparity between Opa1 and Mgm1. First we elaborate on their monomeric forms before discussing oligomeric forms.

The G‐domain of Opa1, unlike most DRPs including Mgm1, contains an additional glycine residue in the G2/switch 1 motif (Fig. 3A), which slows GTPase activity (Yu *et al*., 2020). As this motif is within the interaction interface between opposing G‐domains, the biochemical consequence of this inserted residue is likely augmented dimerization (Nyenhuis *et al*., 2023; Yu *et al*., 2020). Furthermore, a unique coiled‐coil domain upstream of the G‐domain represents an Opa1‐specific dimerization interface lacking in other DRPs (Nyenhuis *et al*., 2023; Zhang *et al*., 2020).

While the stalk domains of Opa1 and Mgm1 are very similar overall, variations to parts of the domain also affect how each DRP's homo‐oligomer is configured (Faelber *et al*., 2019; Nyenhuis *et al*., 2023; von der Malsburg *et al*., 2023; Yan *et al*., 2020). Contrary to Mgm1 and conventional DRPs, two helices of the Opa1 stalk are kinked. Furthermore, Opa1 features a specific loop between stalk α‐helices 2 and 3 that is absent in Mgm1. This loop contributes to a unique interaction interface between the stalk and PD to facilitate dimerization (i2 in Fig. 3A,B; Nyenhuis *et al*., 2023). Mgm1's stalk also has a unique loop between α‐helices 1 and 2 that restricts PD movement (Faelber *et al*., 2019).

Mgm1's PD has three α‐helices, while Opa1's PD is more elaborated and contains six α‐helices (Faelber *et al*., 2019; Nyenhuis *et al*., 2023; von der Malsburg *et al*., 2023; Yan *et al*., 2020) (Fig. 3A). The PD of Opa1 is better adapted for IM interactions than is that of Mgm1. Two Opa1‐specific α‐helices at the stalk/PD hinge fortify IM contact, and a critical membrane insertion loop (MIL) is embedded in the membrane's outer leaflet, that recognizes cardiolipin. Conformational changes involving shifting the PD relative to the stalk pull the MIL from the membrane, possibly extracting the associated cardiolipin, and resulting in packing defects in the outer leaflet that mechanically buckle the membrane. By contrast, the Mgm1 PD contains a glycine‐rich loop (Fig. 3A) that likely interacts superficially with the IM's outer leaflet. Thus, how each DRPs interacts with the IM differs.

### (3) Oligomeric structure

Both S‐Opa1 and S‐Mgm1 form multimeric lattices with helical arrangements along phospholipid tubules, as shown by cryogenic electron microscopy (Faelber *et al*., 2019; Nyenhuis *et al*., 2023; von der Malsburg *et al*., 2023). A rung of each assembly consists of a tetrameric arrangement of two symmetric heterodimers, a structural unit referred to as a protomer (Yan *et al*., 2020). Crystalized S‐Mgm1 from the thermophilic fungus *Chaetomium thermophilum* also assembles into the conventional dimers and tetramers (Faelber *et al*., 2019). Unusually, *S. cerevisiae* S‐Mgm1, appears to assemble into trimers in a crystal lattice (Yan *et al*., 2020), although this trimeric Mgm1 arrangement may represent a crystallization artifact.

Human Opa1 protomers have been observed to assemble into two types of higher‐order oligomers (Fig. 3D). The arrangement we call “Type 1” has been observed in three independent studies (Nyenhuis *et al*., 2023; Yu *et al*., 2020; Zhang *et al*., 2020), and replicated in the nucleotide‐bound and ‐unbound (i.e. apo) lattice structures of *C. thermophilum* Mgm1 (Faelber *et al*., 2019). “Type 2” was solved at high resolution in only one study (Nyenhuis *et al*., 2023), and perhaps also at low resolution (Zhang *et al*., 2020), but is an arrangement never reproduced in Mgm1.

The Type 1 Opa1 oligomer is the more loosely packed of the two assemblies (Fig. 3D), occurring when every Opa1 monomer binds GTP. It is constructed *via* three interaction interfaces: (*i*) an intra‐dimer connection between two monomers; (*ii*) an interface between two dimers to assemble the tetrameric protomer; and (*iii*) an inter‐rung connection between two adjacent protomers. The last of these interfaces is a homotypic interaction formed by the tips of two PD domains in both Mgm1 and Opa1 lattices. Differences in the PDs of Opa1 and Mgm1, the most variable regions of DRPs, result in a discernible ~33 Å shift in rung alignment between the Mgm1 and Opa1 lattice structures (Fig. 3D), which may have functional consequences.

Type 2 is a more tightly packed arrangement observed when each Opa1 monomer is either in an apo or GDP‐bound state, with minor differences between them (Nyenhuis *et al*., 2023). This configuration involves compacted protomers with each monomer's PD and stalk domains highly intertwined, yielding “interlocked dimers” (Nyenhuis *et al*., 2023). The Type 2 lattice is built by nine interaction interfaces, involving elements throughout the DRP (N‐proximal coiled coil, G‐domain, BSE, stalk and PD) that are mostly absent in Mgm1 (Fig. 3) (Nyenhuis *et al*., 2023), another sign that the two DRPs remodel membranes by different mechanisms. It was proposed that the Type 2 higher‐order structure represents a pre‐fusion IM intermediate just before membrane fusion occurs (Nyenhuis *et al*., 2023).

While both homo‐oligomers assume helical arrangements, how these higher‐order structures remodel the IM upon GTP hydrolysis differs greatly. The Mgm1 oligomer structure suggests that it expands and contracts membranes *via* the classical power‐stroke mechanism seen in dynamin and other DRPs (Faelber *et al*., 2019); the more rigid PD domain may provide a strong clamp for this action. By contrast, Opa1 oligomers form a scaffold that brings the two MIL‐buckled IMs in close opposition, making their fusion energetically favourable (Nyenhuis *et al*., 2023). Thus, the significant divergence in Opa1 and Mgm1 primary structure detected by molecular phylogenetics (Sheikh *et al*., 2023) is manifested in distinctive tertiary and quaternary structures.

### (4) Protein interaction networks

Given the role of both in mitochondrial morphogenesis, Mgm1 and Opa1 engage in binary interactions with several other mitochondrial membrane proteins. However, their respective interaction networks do not coincide, indicating their likely independent evolutionary trajectories in Holozoa and Holomycota, respectively, and contributing to their different mechanisms of action.

Opa1 has been reported to interact with several subunits of the mitochondrial contact site and cristae organizing system (MICOS) complex (Patten *et al*., 2014; Ritam *et al*., 2023; Darshi *et al*., 2011), especially its keystone subunit Mic60 (Darshi *et al*., 2011; Barrera *et al*., 2016; Glytsou *et al*., 2016; Schweppe *et al*., 2017) (Fig. 2B). MICOS is an ancient complex derived from the proto‐mitochondrial symbiont (Muñoz‐Gómez *et al*., 2023), repurposed for the formation of crista junctions (CJs), narrow necks that attach cristae to the IM (Wollweber, von der Malsburg & van der Laan, 2017; Colina‐Tenorio *et al*., 2020). Furthermore, Opa1 and Mic10, another core MICOS subunit (Wollweber *et al*., 2017; Colina‐Tenorio *et al*., 2020), act synergistically to stabilize tubular CJs (Stephan *et al*., 2020). By contrast, no interaction between MICOS and Mgm1 has been reported (Wollweber *et al*., 2017; Colina‐Tenorio *et al*., 2020). Moreover, these two entities are not epistatic, implying little functional interplay between them (Hoppins *et al*., 2011). This contrasts with Opa1 being epistatic to Mic60 (Glytsou *et al*., 2016). Consistently, holozoan Mic60 seems to possess a proline‐rich, Opa1‐interacting motif just upstream of its TMD (Schweppe *et al*., 2017) (see online Supporting Information, Fig. S1), suggesting co‐evolution between Mic60 and Opa1. Other metazoan‐specific Mic60 sequences may also represent adaptations to Opa1's enrichment in cristae (Glytsou *et al*., 2016).

Mgm1 contacts the OM by interacting with Fzo1 (Meeusen *et al*., 2006), a DSP more distantly related to Mfn1 and Mfn2 than expected (see Section III.4.a). This indirect interaction is mediated by the mitochondrial carrier protein (MCP) superfamily protein Ugo1 (Sesaki *et al*., 2003; Wong *et al*., 2003) (Fig. 2B). Their epistatic relationship further supports their functional interplay (Hoppins *et al*., 2011). Coincidentally, Opa1 also interacts with a MCP‐like OM protein named SLC25A46 (Janer *et al*., 2016), which is not orthologous to Ugo1 despite their ostensible similarity (Section III.4.a). Nevertheless, a physical interaction between Opa1 and either mitofusin has never been observed (Pernas & Scorrano, 2016).

#### (a) The independent origins of OM interactors of Opa1 and Mgm1

Paralleling the lack of common ancestry of Opa1 and Mgm1 at the root of opisthokonts, there is strong evidence that their OM protein interactors have also been recruited independently. For instance, the DSPs that mediate OM fusion in yeast and vertebrates, Fzo1 and the mitofusins, respectively, exhibit such a high degree of sequence divergence that they appear as closely related to bacterial dynamin‐like proteins as they are to each other *via* current phylogenetic methods (Mattie *et al*., 2017). This challenges the assumption that they share common ancestry within Opisthokonta.

The lack of orthology between vertebrate and yeast OM DSPs is also reflected in their different topologies. Fzo1 has two TMDs that place both of its termini in the cytosol (Mattie *et al*., 2017). By contrast, mitofusins contain a single TMD, and their C‐terminal domains extrude into the IMS. The C‐terminal domain contains a redox‐sensitive motif absent in Fzo1 (Mattie *et al*., 2017). Remarkably, Opa1 likewise has IMS‐localized redox‐sensing elements within its stalk and PD that regulate fusion and crista remodelling (Semenzato *et al*., 2023). Because there is no detectable physical interaction between the mitofusins and Opa1 (Pernas & Scorrano, 2016), it is tempting to speculate that these redox sensors enable coordination of their respective fusion activities (Fig. 2B).

Only a tiny loop between the TMDs of Fzo1 extends into the IMS, perhaps explaining the recruitment of polytopic Ugo1, a member of the MCP superfamily, to link Fzo1 to Mgm1 (Sesaki *et al*., 2003; Wong *et al*., 2003) (Fig. 2B). Opa1 has also been reported to bind the MCP‐like SLC25A46, reminiscent of Mgm1's interaction with Ugo1. However, the two proteins are very distantly related within the MCP superfamily, indicating an independent origin (Muñoz‐Gómez *et al*., 2015; Abrams *et al*., 2015). Ugo1 is restricted to Holomycota and SLC25A46 to vertebrates; its potentially wider distribution in other holozoans has still not been investigated. Further evidence of their independent origin is their opposing mitochondria‐dynamics phenotypes, with *ugo1* deletion phenocopying the fusion defects of *mgm1* (Sesaki *et al*., 2003; Hoppins *et al*., 2009), whereas *SLC25A46* depletion or null mutants exhibit hyperfused mitochondria (Abrams *et al*., 2015; Janer *et al*., 2016). Their opposing role in mitochondrial dynamics is supported by SLC25A46's failure to complement *ugo1* deletion (Abrams *et al*., 2015). Finally, SLC25A46 and the MICOS complex interact and appear to be interdependent (Abrams *et al*., 2015; Janer *et al*., 2016), whereas Ugo1 does not engage in such interplay (Hoppins *et al*., 2011).

In conclusion, the OM interactors with Opa1 and Mgm1 are likely phylogenetically distant players in mitochondrial dynamics, just as are the IM DRPs. This further supports our hypothesis that distinct modes of mitochondrial dynamics emerged in Holozoa and Holomycota, perhaps having a broader impact on the organisms that comprise these two opisthokontan groups.

### (5) IM fusion mechanisms

Differences in processing, structure and protein‐interaction networks hint that Opa1 and Mgm1 use different mechanisms to mediate fusion. Indeed, they differ not only in how they catalyse IM fusion but also in how they coordinate their action with OM fusion.

As confirmed by solved structures, the MIL motif of Opa1 interacts strongly with cardiolipin through intermolecular ionic bonds and intercalation (Nyenhuis *et al*., 2023; von der Malsburg *et al*., 2023) (Fig. 2C). Cardiolipin inserted in one bilayer and L‐Opa1 embedded in the other are the minimal requirements for membrane fusion (Ban *et al*., 2017; Song *et al*., 2009). However, homotypic L‐Opa1 interactions promote the tethering of the two opposing membranes to augment fusion. S‐Opa1 appears to enhance L‐Opa1 binding, regulate IM fusion (Ban *et al*., 2017; Wang *et al*., 2020) and stimulate mitochondrial fission on its own (Anand *et al*., 2014).

By contrast, both juxtaposed IM membranes must contain Mgm1 for their fusion to take place (Meeusen *et al*., 2006; Sesaki *et al*., 2003) (Fig. 2C). Cardiolipin plays an auxiliary role in yeast mitochondrial fusion by stimulating the GTPase activity of Mgm1 (DeVay *et al*., 2009), instead of being directly recruited to mediate fusion as in Opa1. Finally, L‐Mgm1 and S‐Mgm1 are indispensable for fusion, with only the latter's GTPase activity being essential (DeVay *et al*., 2009; Zick *et al*., 2009). While the essentiality of the GTPase activity of S‐Opa1 has not been investigated, this activity is required by L‐Opa1 for IM fusion (Ban *et al*., 2017). In conclusion, Opa1 and Mgm1 mediate IM fusion by very different mechanisms.

**Fig. 4:**
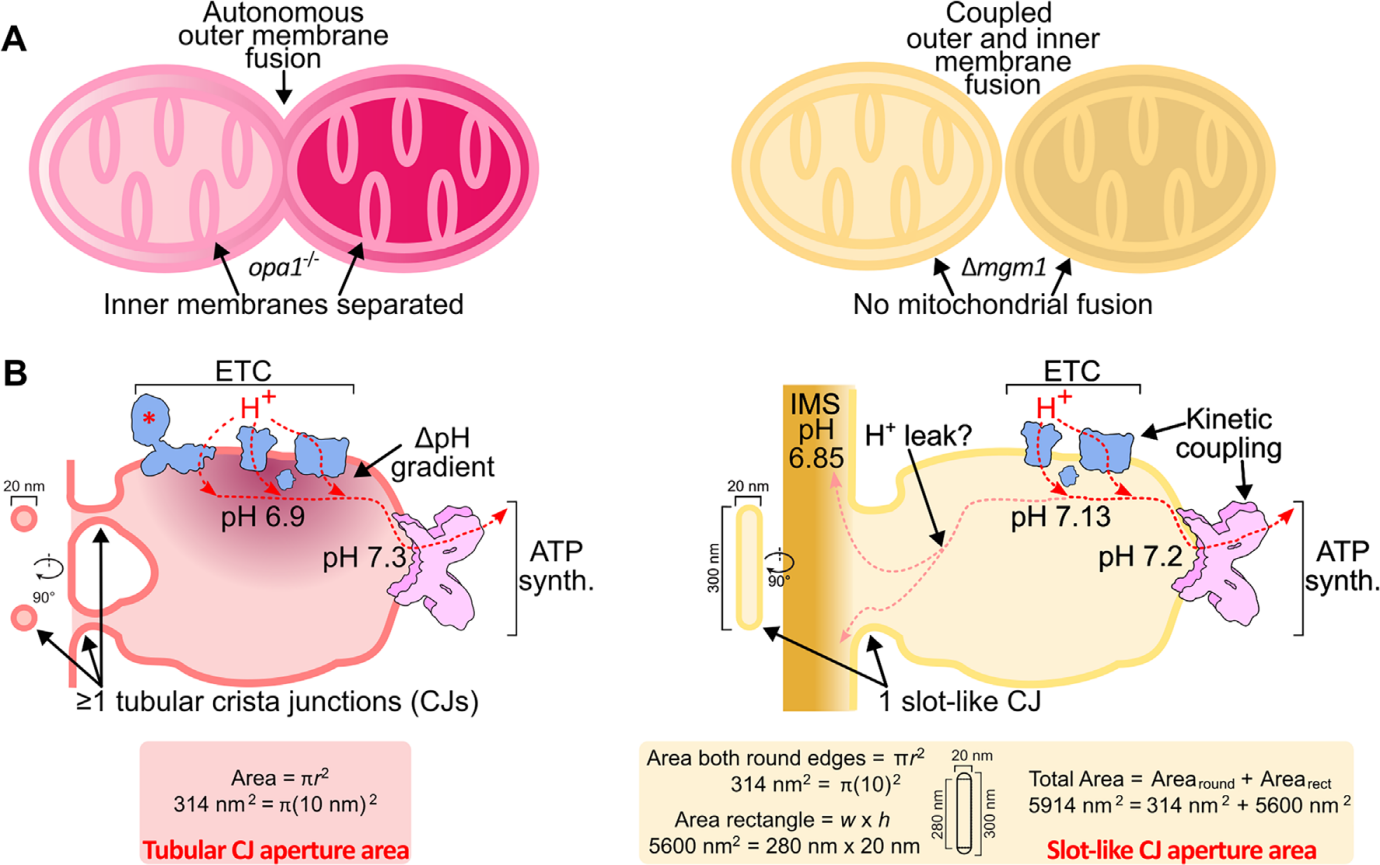
Differences in inner membrane fusion and remodelling in vertebrates (left) and yeast (right). (A) Comparison of *opa1* and *mgm1* null mutations that demonstrate the decoupling of outer and inner membrane fusion in vertebrates and their interconnection in yeast. (B) Differences in vertebrate and yeast crista junction (CJ) morphology may impact how the electron transport chain (ETC) is coupled with ATP synthase (ATP synth.). Simple models to calculate CJ aperture areas are provided in the coloured boxes below. Vertebrate and yeast intracrista pH values are from Rieger *et al*. (2021) and Toth *et al*. (2020), respectively. Note that NADH dehydrogenase (*) is absent in *Saccharomyces cerevisiae* crista. IMS, intermembrane space.

Complete fusion of yeast (Sesaki *et al*., 2003) and vertebrate (Cipolat *et al*., 2004; Song *et al*., 2009) mitochondria requires the coordination of OM and IM fusion apparatuses. However, comparison of Opa1 and Mgm1 ablation phenotypes reveals fundamental differences in OM and IM fusion coupling (Fig. 4A). OM fusion still occurs in *opa1* null mutants, manifested by a single OM surrounding multiple IM‐bound matrices (Song *et al*., 2009). In *mgm1*‐deletion yeast, mitochondrial fusion involving both membranes is halted, resulting in a highly fragmented network (Sesaki *et al*., 2003). The integration of OM and IM fusion is likely due to Mgm1's stable interaction with the OM DSP Fzo1 *via* Ugo1 (Sesaki *et al*., 2003; Wong *et al*., 2003; Fig. 2B), which functionally links them (Hoppins *et al*., 2011).

### (6) mtDNA maintenance

Mitochondrial fusion is fundamental for mtDNA inheritance (Pernas & Scorrano, 2016). Thus, both Opa1 and Mgm1 invariably are involved in mtDNA upkeep, albeit to different extents (Elachouri *et al*., 2011; He *et al*., 2022; Jones & Fangman, 1992; Sesaki *et al*., 2003). Indeed, only Opa1 isoforms containing the exon 4b‐encoded polypeptide appear to mediate mtDNA maintenance (Elachouri *et al*., 2011). Given that these isoforms are entirely processed into short forms (Wang *et al*., 2020), it was proposed that the N‐terminal part downstream of the S3 cleavage site may persist after endoproteolysis to anchor mtDNA to the IM (Elachouri *et al*., 2011) (Fig. 2A). Consistently, it has been reported that this exon4b‐encoded fragment may bind to the mtDNA D‐loop (Yang *et al*., 2020), a three‐stranded DNA structure that is needed for mtDNA replication and expression (Nicholls & Minczuk, 2014). However, exon 4b‐derived peptide most likely would be comprised of an IMS‐extruding loop in between two TMDs (Fig. 2A), which is a topology not conducive for binding matrix‐localized mtDNA. More work is needed to pinpoint Opa1's exact – and possibly moonlighting – role in mtDNA maintenance.

A forward genetics screen for proteins responsible for mtDNA inheritance led to the discovery of Mgm1, reflected in its original moniker (Jones & Fangman, 1992). However, it was later shown that Mgm1's role is indirect, as the cessation of mitochondrial fusion upon *mgm1* deletion led to secondary loss of mtDNA (Sesaki *et al*., 2003). This was elegantly demonstrated in yeast in which *dnm1* and *mgm1* were simultaneously deleted (Δ*dnm1*Δ*mgm1*), the former encoding the mitochondrial fusion DRP. This genotype retained mtDNA by preserving a fused – albeit static – mitochondrial network. In conclusion, Opa1 and Mgm1 mediate mtDNA maintenance in different ways, with the former possibly having a still ambiguous dedicated role whereas the role of the latter is an indirect consequence of being a major player in yeast mitochondrial dynamics.

### (7) Crista remodelling

IM morphogenesis also includes the reshaping of cristae, the seats of cellular respiration within the organelle (Pánek *et al*., 2020; Wolf *et al*., 2019). Both Opa1 and Mgm1 have been implicated in this process based on gene ablation. However, a closer comparison of the resulting phenotypes indicates that they mediate crista remodelling differently (Fig. 2D).

Acute mRNA depletion (Frezza *et al*., 2006; Griparic *et al*., 2004; Olichon *et al*., 2003; Stephan *et al*., 2020), conditional gene knockout (Glytsou *et al*., 2016; Cogliati *et al*., 2013), GTPase loss of function (Frezza *et al*., 2006), and heterozygous null mutations (Barrera *et al*., 2016) of *opa1* developed enlarged cristae (i.e. crista membranes enclosing a wider space), sometimes accompanied by broader CJs (Glytsou *et al*., 2016; Frezza *et al*., 2006; Stephan *et al*., 2020; Fry *et al*., 2024; Suga *et al*., 2023). The width of cristae and their junctions, plus other morphological parameters, are fine‐tuned by the L‐Opa1:S‐Opa1 ratio (Lee, Smith & Yoon, 2017; Fry *et al*., 2024). Consequently, Opa1 second‐handedly regulates respiratory chain supercomplex formation by shaping cristae (Cogliati *et al*., 2013). This may explain why *opa1* missense and heterozygous null mutants exhibit excessive reactive oxygen species (ROS) production (Del Dotto & Carelli, 2021).

Mitochondria of *mgm1*‐deletion mutants observed by thin‐section transmission electron microscopy contain fewer cristae, which are usually deformed (Meeusen *et al*., 2006; Sesaki *et al*., 2003). Given these phenotypes are confounded by the aforementioned mtDNA loss, more insight can be gained from temperature‐sensitive *mgm1* mutants (*mgm1* ^ts^) that lose function only at elevated temperature (Meeusen *et al*., 2006). During a short heat‐shock pulse, *mgm1* ^ts^ lose all lamellar cristae prior to losing their mtDNA (Harner *et al*., 2016). Interestingly, narrower tubular cristae predominate under this condition, which is contrary to the prevailing intracristal‐space expansion from induced *opa1* ablation. Furthermore, CJs and respiratory chain supercomplex formation are not affected at elevated temperature, perhaps due to the persistence of tubular cristae during heat shock. Consistent with the latter, *mgm1* mutants do not exhibit elevated ROS levels (Del Dotto & Carelli, 2021). The loss of lamellar cristae was phenocopied in Δ*dnm1*Δ*mgm1* yeast, wherein both fission and fusion are suppressed, leading to the conclusion that lamellar cristae biogenesis relies on Mgm1‐mediated IM fusion (Harner *et al*., 2016), a hypothesis corroborated later (Kojima *et al*., 2019).

In summary, while Opa1 and Mgm1 exhibit some convergent properties as DRPs that are post‐translationally processed and remodel the IM in ostensibly similar ways, they exhibit fundamental differences in how they carry out these roles. These roles are inherent in their structural divergence, likely due to their independent origins in the respective cenancestors of Holozoa and Holomycota. Thus, they possibly underwent independent evolution in these two opisthokontan lineages, resulting in fundamentally divergent impacts on IM morphogenesis.

## IV. DIFFERENCES IN OPA1 AND MGM1 MAY ENDOW ANIMALS AND FUNGI WITH FUNDAMENTALLY DIFFERENT MITOCHONDRIAL DYNAMICS AND PHYSIOLOGY

The mitochondria of animals and fungi seemingly look and act alike. For example, they both contain plate‐like, lamellar cristae, once considered a morphological synapomorphy that unifies opisthokonts (Pánek *et al*., 2020). While both mitochondria are shaped by seemingly very similar IM DRPs, molecular phylogenetics suggests they originated independently (Sheikh *et al*., 2023). The enumerated disparities between Opa1 and Mgm1 can be most parsimoniously explained by their different evolutionary origin. But what are the possible ramifications of these differences for mitochondrial dynamics in metazoans and fungi? And how could they ultimately impact the fundamental biology of these lineages?

### (1) The decoupling of OM and IM fusion unleashes dynamic cristae in vertebrates

Unlike fungi, OM and IM fusion in vertebrates occur autonomously (Fig. 4A). This is clearly demonstrated by the lack of physical interaction between the OM and IM fusion apparatuses, and correlates with the persistence of OM fusion when IM fusion is inhibited by Opa1 depletion. Since Opa1 is disconnected from OM fusion and can directly remodel cardiolipin‐containing membranes, the DRP is free to sculpt already extant cristae. Opa1 is not the sole factor in lamellar cristae biogenesis, but rather shapes these ultrastructures (Stephan *et al*., 2020; Fry *et al*., 2024).

Remarkably, crista dynamics is not restricted to their contraction and expansion, shortening and elongation. Like the organelles that contain them, vertebrate cristae are also able to fuse and divide in a MICOS‐ and Opa1‐dependent manner (Kondadi *et al*., 2019; Hu *et al*., 2020), although mtDNA loss in *opa1* knockouts has not been excluded as the cause of abnormalities in crista dynamics (Hu *et al*., 2020). Crista fusion may also explain why volumetric‐electron micrographs of fixed vertebrate mitochondria reveal multiple CJs emanating from lamellar cristae (Pánek *et al*., 2020); these may be captured fused cristae, sometimes occurring between CJs as they can have a slot‐like instead of the typical tubular morphotype (Kondadi *et al*., 2019; Hu *et al*., 2020). Furthermore, cristae also can detach and reattach to the IM. Thus, vertebrates – and perhaps metazoans or holozoans in general – have particularly dynamic cristae. What is the biological outcome of this trait?

### (2) Dynamic cristae pave the way for cytochrome c‐mediated apoptosis

Programmed cell death by apoptosis is essential for metazoan development (Elmore, 2007). Apoptosis is initiated by extracellular or intracellular signals to start extrinsic or intrinsic apoptosis, respectively. Both pathways activate procaspase 3 into a protease that executes apoptotic events like DNA fragmentation and membrane blebbing.

Mitochondria play a central role in initiating intrinsic or amplifying extrinsic apoptosis signalling pathways. The proapoptotic factor tBid, an activated form of the zymogen BH3 interacting‐domain death agonist (Bid), contacts the OM to stimulate the organelle's involvement in the process. Mitochondrial outer membrane permeabilization (MOMP) occurs during vertebrate apoptosis, releasing cytochrome c into the cytosol, where it binds the apoptotic protease release factor Apaf‐1. In this bound state, Apaf‐1 heptamerizes into the apoptosome, which ultimately induces a protease cascade that sequentially activates caspase 9 and eventually caspase 3.

In living cells, cytochrome c is a soluble component of the otherwise crista‐membrane‐embedded electron transport chain (ETC) (Pánek *et al*., 2020; Wolf *et al*., 2019). Thus, cytochrome c is normally sequestered within the intracristal space (Scorrano *et al*., 2002). Given the involvement of cytochrome c in apoptosis, Opa1 has come to play an important regulatory role in apoptosis (Olichon *et al*., 2003; Frezza *et al*., 2006; Cipolat *et al*., 2006; Fry *et al*., 2024; Merkwirth *et al*., 2008; Yamaguchi *et al*., 2008). By remodelling cristae, Opa1 releases cytochrome c into the IMS so it can percolate into the cytosol *via* MOMP (Olichon *et al*., 2003; Frezza *et al*., 2006; Cipolat *et al*., 2006; Fry *et al*., 2024; Merkwirth *et al*., 2008) (Fig. 2D). An alternative proposed mechanism is that Opa1 homo‐oligomers cage cytochrome c, which is expelled from cristae upon Opa1 depolymerization (Yamaguchi *et al*., 2008).

However, the idea that Opa1 plays an essential role in apoptosis in all metazoans is questioned by cytosolic cytochrome c not being essential in two‐thirds of the animal models where apoptosis is most studied. Below, we provide arguments to resolve this apparent contradiction, preserving the possibility that dynamic cristae may be a necessary trait for the emergence of apoptosis in Metazoa.

#### (a) The many faces of death in metazoans: the various roles of mitochondria in apoptosis

**Fig. 5:**
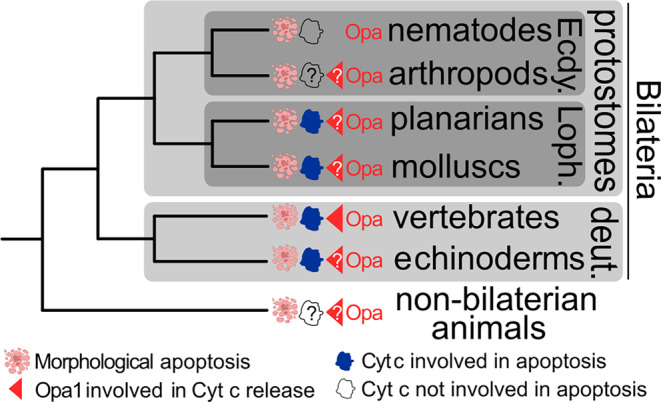
The distribution of cytochrome c‐mediated apoptosis in metazoans. Cyt c, cytochrome c; deut., deuterostomes Ecdy., Ecdysozoa; Loph., Lophotrochozoa; Opa 1, optical atrophy 1.

Ecdysozoa is a metazoan taxon that contains two out of the three most studied animal models of apoptosis: the nematode *Caenorhabditis elegans*, where many molecules involved in apoptosis were first discovered, and the fruit fly *Drosophila melanogaster*, an arthropod (Fig. 5). However, MOMP and cytochrome c induction of apoptosome assembly are not essential for apoptosis in these organisms, giving the impression that the involvement of these events only emerged in vertebrates (Hofmann, 2020; Oberst, Bender & Green, 2008).

In *C. elegans*, the absence of involvement of cytosolic cytochrome c is evident (Fig. 5), with its Apaf‐1 ortholog having lost the ability to bind cytochrome c (Hofmann, 2020; Oberst *et al*., 2008). In accordance, Opa1 is also not involved in nematode apoptosis (Del Dotto & Carelli, 2021; Kanazawa *et al*., 2008). This peculiarity may also be connected to the ortholog's loss of stress‐induced Oma1 cleavage and its considerably reduced isoform complexity, with Oma1 existing as one S‐form and one L‐form (Chaudhari & Kipreos, 2017). Furthermore, the reduction of a putative Opa1‐interaction interface in *C. elegans* Mic60 paralogs (Fig. S1) suggests that interaction of these two proteins is needed for CJ remodelling. The situation in *D. melanogaster* is less clear, but MOMP and cytosolic cytochrome c seem to be expendable for the initiation of intrinsic and extrinsic apoptosis (Oberst *et al*., 2008). The role of Opa1 in fruit fly apoptosis is also ambiguous (Yarosh *et al*., 2008), in contrast to the clear role of vertebrate Opa1 in this process (Olichon *et al*., 2003; Frezza *et al*., 2006; Cipolat *et al*., 2006; Fry *et al*., 2024; Merkwirth *et al*., 2008; Yamaguchi *et al*., 2008).

The lack of involvement of cytosolic cytochrome c in two of the best studied models of apoptosis seems to contradict our thesis that Opa1‐mediated IM dynamics represents a necessary pre‐adaptation towards metazoan multicellularity, as apoptosis is also a vital part of nematode and *D. melanogaster* development (Elmore, 2007). However, there is evidence that cytochrome c is involved in apoptosis in Echinodermata (e.g. sea urchins and sand dollars) and Lophotrochozoa (e.g. planarians and molluscs) (Bender *et al*., 2012; Li *et al*., 2017). Thus, cytochrome c‐dependent apoptosis may have been an ancestral trait of at least Bilateria as it is observed in representatives of both its major subdivisions: Deuterostomia, encompassing vertebrates and echinoderms, and Protostomia, which includes Lophotrochozoa and Ecdysozoa (Fig. 5). The involvement of cytosolic cytochrome c in apoptosis is more likely to have been secondarily lost in ecdysozoans than to have emerged in four separate metazoan taxa independently. Furthermore, the majority of molecules that mediate apoptosis are shared by all of these organisms, implying a single origin of apoptosis in bilaterians, with only some losses (e.g. Apaf‐1 in *C. elegans*) (Hofmann, 2020; Oberst *et al*., 2008).

The theory that MOMP and cytochrome c release were original traits of apoptosis makes two testable predictions. The first is that cytochrome c release is an important factor for apoptosis in non‐bilaterians such as cnidarians (e.g. jellyfish), placozoans (extremely simplified, blob‐like animals), ctenophoreans (comb jellies), and poriferans (e.g. sea sponges). The second is that Opa1, a protein found in all holozoans (Sheikh *et al*., 2023), mediates cytochrome c release in the taxa where this is an integral step in intracellular apoptotic signalling.

### (3) Fusion is coupled to lamellar crista biogenesis in fungi

Unlike vertebrates, OM and IM fusion are inseparable in fungi. Mgm1 physically interacts with the OM fusion factors Ugo1 and Fzo1. When IM fusion is inhibited by *mgm1* deletion, OM fusion ceases.

The necessity for Mgm1‐mediated mitochondrial fusion for lamellar cristae biogenesis (Fig. 2D) in Holomycota could be a consequence of the linkage between the IM and OM. Contrasting with Opa1's crista‐shaping activity, Mgm1 appear to be the sole factor responsible for lamellar cristae formation in fungi (Harner *et al*., 2016; Kojima *et al*., 2019). This may also explain why fungal cells exhibit more frequent mitochondrial‐dynamics events – represented equally by fission and fusion (Pernas & Scorrano, 2016) – than do vertebrate cells. While a parallel comparison has never been carried out to our knowledge, such events occur approximately every 2 min in budding yeast (Nunnari *et al*., 1997), an order of magnitude more often than in immortalized cell lines originating from monkeys (~20 min) (Twig *et al*., 2008) and rats (~70 min) (Di Meo *et al*., 2021). Thus, rapid mitochondrial fusion may be an adaptation for lamellar cristae biogenesis in fungi. By contrast, crista dynamics in animals may maintain lamellar cristae to compensate for the relative infrequency of mitochondrial fusion.

Another consequence of interlinked OM and IM fusion in yeasts is that the fusion activity of Mgm1 cannot be decoupled from its role in crista biogenesis. Indeed, Mgm1 only superficially binds cardiolipin‐enriched membranes (DeVay *et al*., 2009), strictly requiring homotypic interactions to fuse membranes (Faelber *et al*., 2019; Sesaki *et al*., 2003; Meeusen *et al*., 2006). Thus, Mgm1 may not be capable of being repurposed in the same way as Opa1, perhaps precluding the emergence of apoptosis‐like programmed cell death in fungi, and maybe even in Holomycota as a whole. Since cytochrome c leakage from mitochondria will not then induce spurious apoptosis in fungi, the selective pressure to maintain tightly constricted CJs may have been relaxed in this lineage. Perhaps this is why yeast CJs are almost exclusively slot‐like, as opposed to the predominant tubular morphology seen in animals (Pánek *et al*., 2020) (Fig. 4B).

### (4) The distinctive cristae junctions of metazoans and fungi may differentially impact electron transport chain–ATP synthase coupling

CJs are proposed to be diffusion barriers that serve to subcompartmentalize cristae (Wolf *et al*., 2019; Pánek *et al*., 2020). Therefore, different CJ morphologies may have impacted how cristae in fungi and animals act as bioenergetic subcompartments (Wolf *et al*., 2019; Pánek *et al*., 2020). Based on the average dimensions of CJs in fungi and animals (Pánek *et al*., 2020; Davies *et al*., 2011), a simplified model suggests that slot‐like CJs are up to ~19 times more expansive than tubular ones (Fig. 4D). However, apart from allowing cytochrome c translocation, how this expansion of CJs affects mitochondrial physiology remains obscure.

Nevertheless, this parameter may help to reconcile two competing theories explaining the function of cristae in oxidative phosphorylation, that is cellular respiration (Rieger, Junge & Busch, 2014; Rieger *et al*., 2021; Toth *et al*., 2020) (Fig. 4D). Both theories agree that cristae spatially organize respiratory chain complexes, with the ETC localizing to flat membrane tracts and ATP synthase enriched within the positive membrane curvature at crista edges (Davies *et al*., 2011). Their partitioning parallels their synergistic roles in oxidative phosphorylation: the electrochemical gradient generated by the ETC is consumed by ATP synthase to produce chemical energy for the cell.

Results from the HeLa cell line indicate that protons accumulated by the ETC into the proximal intracristal space flow laterally to ATP synthase located at the tips, which acts as a proton sink (Rieger *et al*., 2021, 2014). Thus, the authors theorized that cristae insulate this lateral electrochemical gradient by mitigating proton leak from the base of cristae. Indeed, expanded CJs dissipate the ETC‐generated electrochemical gradient in assorted human cell lines (Wolf *et al*., 2019). An equivalent lateral pH gradient was not detected within *S. cerevisiae* cristae, prompting the alternative hypothesis that cristae only serve to keep the ETC and ATP synthase together to couple them kinetically (Toth *et al*., 2020).

One explanation for this discrepancy is that *S. cerevisiae* lacks NADH dehydrogenase (i.e. respiratory complex I) (Pagliarini *et al*., 2008), which normally contributes a significant portion of the proton pumping capacity of the ETC; this could be one reason for the steeper lateral pH gradient seen in HeLa cells (Fig. 4D). Another explanation lies in the different CJs in each organism. The mathematical model used to interpret the HeLa‐cell results assumed obstructed CJs (Rieger *et al*., 2014), a proxy for their real role as diffusion barriers (Wolf *et al*., 2019). By contrast, the slot‐like CJs in yeast could be more susceptible to proton leak, ultimately short circuiting a nascent lateral pH gradient. In agreement with this, inhibition of ATP synthase, which acidifies the confined intracrista space of humans (Rieger *et al*., 2021), did not have such an effect in yeast (Toth *et al*., 2020). This can be explained by a relatively higher permeability of wider slot‐like CJs in yeast. Furthermore, the IMS between the two mitochondrial membranes is enigmatically more acidic than the intracrista space, suggesting that proton leak through slot‐like CJs occurs at a steady state. In conclusion, the different crista architectures of animals and fungi may also mechanistically influence the bioenergetic capacity of their respective mitochondria.

## V. WHAT WAS THE ORIGINAL OPISTHOKONTAN MITOCHONDRIAL IM‐SHAPING DYNAMIN‐RELATED PROTEIN?

In opisthokontan mitochondria, the IMs of its two subdivisions, Holozoa and Holomycota, are sculpted by DRPs that share certain properties, perhaps correlated with their equivalent IM architecture (Pánek *et al*., 2020). What hypothetical evolutionary scenario can explain the mutually exclusive distribution of Opa1 and Mgm1 in Holozoa and Holomycota?

It is possible that Opa1 and Mgm1 are both derived from a single ancestral DRP. In this scenario, Opa1 and Mgm1 evolved into the diverged forms that display the significant differences summarized herein. However, phylogenetic analyses do not support this hypothesis, although it should be noted the highly divergent nature of the examined DRPs precludes any confident conclusions about their phylogeny (Sheikh *et al*., 2023). Alternatively, both DRPs could have evolved in parallel because of gene duplication in an opisthokontan ancestor followed by reciprocal gene loss in each subdivision.

Another scenario is that one of the two proteins evolved first and was subsequently replaced by the other after the split of Holomycota and Holozoa. For example, let us speculate that Opa1 is the older of the two opisthokontan IM DRPs, and was replaced by Mgm1 in the holomycotan cenancestor. This hypothesis is supported by presence of *opa1* in two representatives of the CRuMs supergroup, which is closely related to Amorphea, the group containing opisthokonts (Sheikh *et al*., 2023) (Fig. 1A). This implies either that *opa1* was present before CRuMs and Amorphea split and was secondarily lost repeatedly, or CRuMs gained *opa1* from a holozoan representative(s) by horizontal gene transfer (HGT), which would invalidate the hypothesis that Opa1 is the older of the two opisthokontan DRPs.

It is worth noting indirect evidence that the role of Opa1 can be substituted in Opisthokonta. The mitochondrial DRP MidX has been identified in a single choanoflagellate species (Sheikh *et al*., 2023), among the closest protist relatives of Metazoa (Ocaña‐Pallarès *et al*., 2022; Ros‐Rocher *et al*., 2021) (Fig. 1A). This almost certainly represents a secondary gain by HGT. Astonishingly, its genome lacks *opa1*, even though this gene is present in all other examined choanoflagellate species (Sheikh *et al*., 2023). We can imagine an analogous scenario where a holomycotan cenancestor acquired *midX* by HGT, then repurposed it to substitute *opa1*, becoming *mgm1*. However, this scenario would invoke a radical transmogrification of MidX that is reflected in currently ambiguous molecular phylogenetics, precluding a definitive answer on the relationship between *midx* and *mgm1* (Sheikh *et al*., 2023).

Until we have more functional data outside the mammalian and yeast models – for example from choanoflagellates that replaced *opa1* with *midX* – and broader taxon sampling of non‐opisthokont Amorphea, we cannot say with high confidence how Opa1 and Mgm1 originated.

## VI. PREDICTIONS AND OPEN QUESTIONS

### (1) Predictions if different IM fusion mechanisms arose in animals and fungi

#### (a) Lamellar crista fission and fusion does not occur in fungi or Holomycota

The physiological role of crista fission and fusion has been proposed to facilitate quality control of respiratory chain complexes, metabolite exchange and/or mtDNA inheritance in the relatively static mitochondrial network of Metazoa (Kondadi, Anand & Reichert, 2020). Indeed, this property may have emerged as an adaptation to this relatively static nature. Conversely, in fungi mitochondria are more dynamic and thus dynamic crista fusion and fission is unnecessary. Indeed, yeast tubular cristae do not appear to agglomerate into the lamellar form. However, it should be noted there are hints that these emergent tubular cristae may moderately fuse with each other (Harner *et al*., 2016).

#### (b) Yeast tubular cristae are an adaptation to compensate for suspended mitochondrial fusion

The physiological advantage of lamellar cristae over other morphotypes (e.g. tubular) remains unknown (Pánek *et al*., 2020). It is possible that the richer mosaic of membrane curvatures may allow better packing and organization of respiratory chain complexes to enable more efficient coupling of the ETC and ATP synthase. We propose that tubular cristae arose to compensate for the depletion of lamellar cristae as a consequence of suspended fusion, which occurs during yeast quiescence (Laporte *et al*., 2018).

### (2) Open questions about mitochondrial dynamics

#### (a) Are there other DSPs, including DRPs, that play a role in IM dynamics?

An IM mitofusin‐like (MfnL) DSP (Fig. 1A) seems to be responsible for branching of mitochondria by an elusive mechanism in trypanosomes (Morel *et al*., 2023; Vanwalleghem *et al*., 2015), which are members of the supergroup Discoba (Hashimi, 2019). MidX, the DRP that is closely related to Mgm1, may also mediate IM fusion and/or other roles in mitochondrial morphogenesis/dynamics. If so, this would be *via* a novel mechanism as MidX is targeted inside the matrix (Sheikh *et al*., 2023).

#### (b) Can molecules other than DSPs and DRPs mediate mitochondrial membrane remodelling?

Recent discoveries in land plants hint at diverse mechanisms that can mediate mitochondrial fusion (White *et al*., 2020). Plants do not have any OM DSP or IM DRP that could serve analogous functions to mitofusin/Fzo1 and Opa1/Mgm1, respectively. Instead, they have recruited a Rho GTPase ortholog of animal Miro and yeast Gem1, which respectively mediate intracellular mitochondria transport and contact‐site formation between mitochondria and the endoplasmic reticulum (Zinsmaier, 2021). Yeast tubular cristae also may undergo modest fusion in a Mgm1‐autonomous manner, albeit without producing extensive lamellar cristae (Harner *et al*., 2016).

#### (c) How do Mgm1 and Opa1 lattices behave in vivo?

Structures of the two S‐forms of Mgm1 and Opa1 were solved in a single state: either GTP‐bound, GDP‐bound or unbound. It is easy to imagine that there will be heterogeneity in the states of the incorporated DRP monomers *in vivo*, perhaps locally modifying the tracts of the helical lattices to modulate remodelling. What is the stoichiometry of L‐forms and S‐forms in these lattices? Can L‐forms alone create helical assemblies in the way that S‐forms do? Are there differences in these lattice behaviours for Mgm1 and Opa1?

## VII. CONCLUSIONS

(1) The distribution of Opa1 and Mgm1 parallels bifurcation of Opisthokonta into Holozoa and Holomycota.
(2) Opa1 and Mgm1 likely did not evolve from a cenancestor, as current molecular phylogenetic methods fail to group them together in one clade. Instead Mgm1 is placed with the novel MidX, which likely has an extra‐opisthokontan origin.
(3) Opa1 and Mgm1 are therefore two significantly different DRPs that have their own biochemical traits and mechanisms for membrane remodelling, resulting from their significant structural differences. The superficial resemblance of the two proteins may be due to convergent evolution.
(4) Co‐evolution of Opa1 and Mgm1 with their respective molecular milieus may wholly or in part underlie fundamental differences in mitochondrial dynamics and crista morphogenesis in animals and fungi. This subsequently could have a broader impact on animal and fungal mitochondrial physiology.
(5) Physiological properties present in animal mitochondria but lacking in fungi may have facilitated in part the emergence of apoptosis in animals. Apoptosis became integrated into the development of complex body plans of animals, which ultimately enabled them to evolve movement and interaction with their surroundings. Without apoptosis, the multicellular fungi are less complex and stationary. In the end, humans hunt for mushrooms, not the other way around.
(6) Our theory discussed in this review would not have been possible without invaluable research on *S. cerevisiae* and other fungal model mitochondria. Comparing the cell biology of animals with that of other eukaryotic lineages can ultimately yield insights into human biology (Hashimi, 2019; Lynch *et al*., 2014).

## Supporting information


**Fig. S1.** Alignment of Mic60 from various opisthokonts reveals a putative optical atrophy 1 (Opa1) interaction interface in Holozoa.
