# Supplementary material for: Bringing together but staying apart: decisive differences in animal and fungal mitochondrial inner membrane fusion
Source: Biol Rev Camb Philos Soc. 2024 Nov 18;100(2):920–35. doi: 10.1111/brv.13168 (PMC11885689; doi:10.1111/brv.13168)
Supplement: Supplementary file 1 — Fig. S1. Alignment of Mic60 from various opisthokonts reveals a putative optical atrophy 1 (Opa1) interaction interface in Holozoa. [file BRV-100-920-s001.pdf]

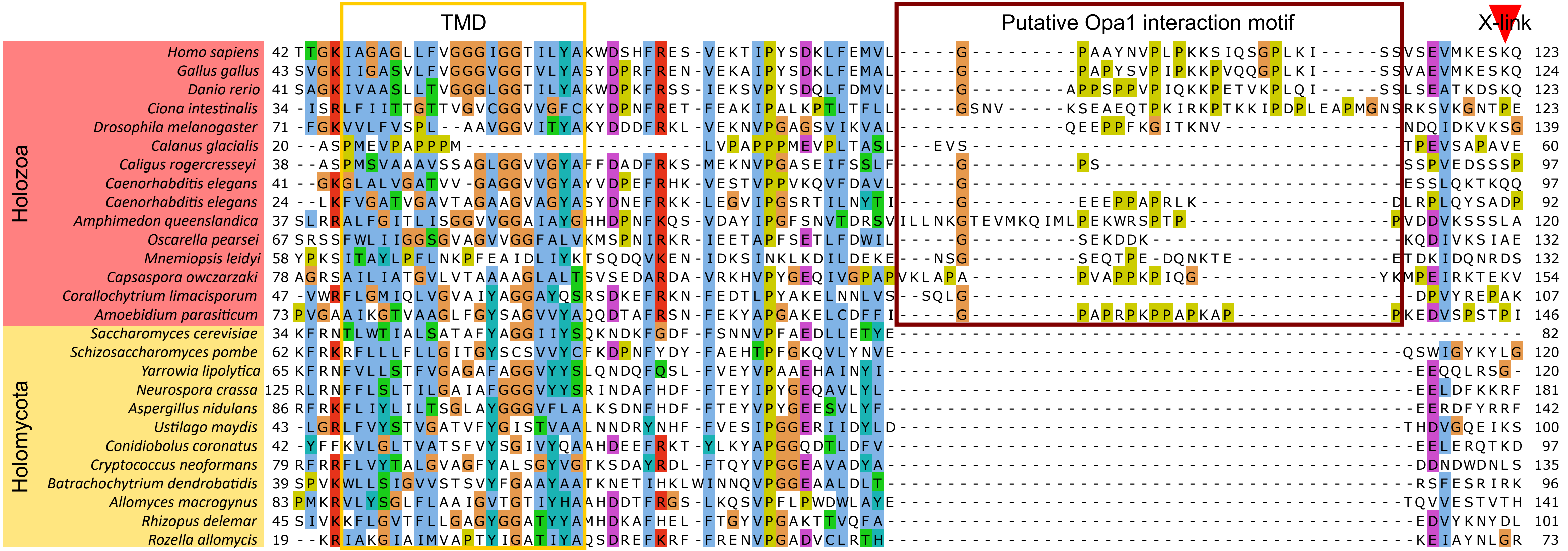

Fig. S1. Alignment of Mic60 from various opisthokonts reveals a putative optical atrophy 1 (Opa1) interaction interface in Holozoa. A portion of Mic60 including its transmembrane domain (TMD; boxed in gold) and cross-linked lysine (X-link) to Opa1 in mouse (Schweppe *et al.*, 2017) aligned by MAFFT (Katoh & Standley, 2013) shows a downstream sequence in holozoans (boxed in brown) that is absent in holomycotans. This sequence rich in proline (P) amino acids may represent a Opa1-interaction interface, which seems to continue to sequence aligning with the X-linked lysine. Also note that this interface is reduced considerably in *C. elegans*, where cytochrome c-mediated apoptosis has been lost.
